# Reply to Alexandre et al.: Insensitivity of the Δ^′17^O value of equisetum to atmospheric relative humidity

**DOI:** 10.1073/pnas.2604539123

**Published:** 2026-04-06

**Authors:** Zachary Sharp, Anthony Gargano, Vincent Hare, Jordan Wostbrock

**Affiliations:** ^a^Department of Earth and Planetary Sciences, University of New Mexico, Albuquerque, NM 87122; ^b^Department of Archaeology, University of Cape Town, Rondebosch 7701, South Africa; ^c^Earth and Planetary Sciences, Yale University, New Haven, CT 06511

Alexandre et al. (1) contest some of the conclusions of our original paper (2), arguing that the Δ^′17^O value of phytoliths is a “quantitative proxy” for relative humidity. We agree that there are conditions where an empirical relationship generally holds, as stated in our original paper—“*there are clear relationships between relative humidity and the triple oxygen isotope values of phytoliths that can be used qualitatively to constrain relative humidity.*” Nevertheless, we contend the Δ^′17^O value of phytoliths is a function of multiple variables, and assigning a linear relationship to relative humidity for our phytolith samples is invalid.

Alexandre et al. (1) calculate RH (%) = 0.21 × Δ^′17^O_phytolith_ (per meg) + 112, based on greenhouse growth experiments at 40, 60, and 80% relative humidity from Outrequin et al. (2). The measured Δ^′17^O_phytolith_ values from our equisetum study range from −232 to −555 per meg (2), corresponding to RH of −4 to 63% using the above equation, clearly not correct. Alexandre et al. (1) use the average Δ^′17^O value of our three phytolith samples as the average for the entire plant, and calculate an RH of 31%, in good agreement with our estimated value of 28%. However, three single samples from a stalk that has a >1,000 per meg range in Δ^′17^O are not necessarily representative of the entire plant.

The average Δ^′17^O for the entire plant should be the sum Δ^′17^O value of each segment. We assume that each segment consists of phytoliths that continually grow as the plant elongates, with large changes in Δ^′17^O of the stem water (2). We determine the instantaneous phytolith Δ^′17^O values by averaging each overgrowth along the length of the plant, equivalent to

$\Delta^{\prime 17}O_{average\;plant\;silica}=\sum_{i=1}^{j}\Delta^{\prime 17}O_{i,silica}$, where $\delta^{\prime 18}O_{i,silica}=\delta^{\prime 18}$ $O_{i,leaf\;water}+\frac{4.28\times 10^{6}}{T^{2}}-\frac{3500}{T}$ (3) and $\delta^{\prime 17}O_{i,silica}=\theta \times \delta^{\prime 18}O_{i,silica}$. *j* is the number of segments considered. The θ value is the empirical fit of 0.5205 from our measured base phytolith value (1).

**Fig. 1.**
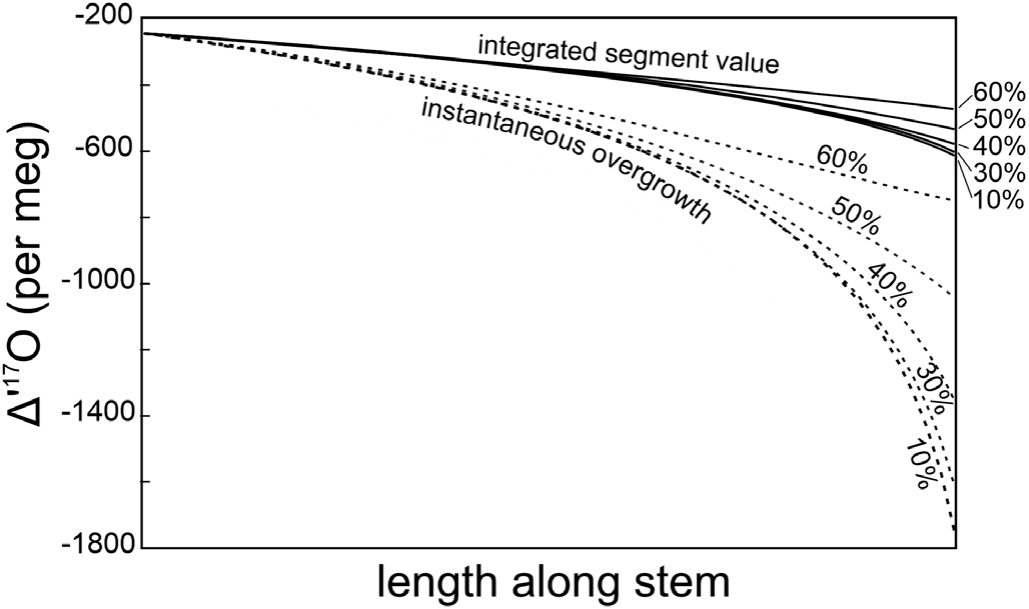
Calculated phytolith Δ^′17^O values for equisetum using growth conditions from Sharp et al. (2) with varying assumed relative humidity (shown by %). The instantaneous overgrowth curve refers to phytolith values in equilibrium with the stem water at that location along the stem. The integrated total segment value assumes that phytoliths form continuously as the plant grows and that each overgrowth forms farther from the base of the plant. We assume all segments contain the same amount of silica for simplicity. We estimate the average silica value for a range of RH values, using the equations for leaf water in equisetum derived in Sharp et al. (2).

**Fig. 2.**
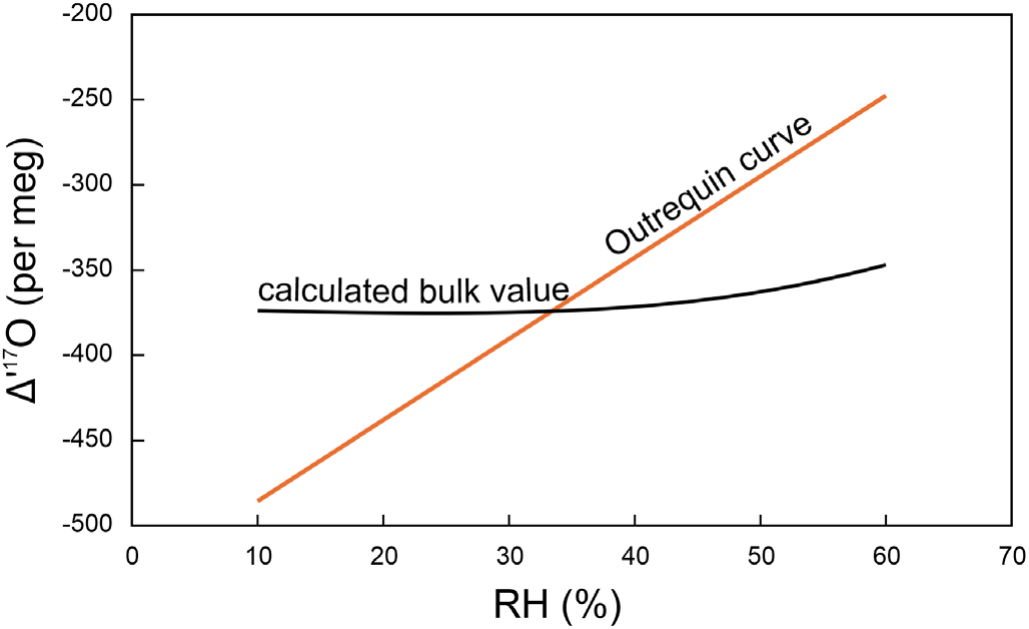
Plot of calculated homogenized (bulk) Δ^′17^O value of an entire plant’s phytoliths as a function of relative humidity (black curve) from Fig. 1 and Outrequin et al. (4) curve for Δ^′17^O vs. RH (orange line). The two curves fortuitously intersect at ~33%, which is close to the relative humidity at our field site.

The calculated outermost (instantaneous) growth band Δ^′17^O value at a given height is shown in Fig. 1, calculated for RH values from 10 to 60%. The bulk value for each phytolith along the stalk length is also shown. While the instantaneous Δ^′17^O value of the phytolith growing at the tip of an equisetum stalk varies by ~1,000 per meg between 10 and 60% relative humidity, the overall integrated value of the entire stalk is surprisingly constant at −379 ± 10 per meg, covering a range of only 28 per meg over an RH range of 10 to 60%. This value fortuitously intersects the Outrequin et al. (4) calibration at ~33% RH (Fig. 2). It is clear, therefore, that the claim that our measured equisetum value reflects the correct relative humidity using the RH-Δ^′17^O equation above (2) is simply fortuitous, and not a reliable, quantitative indicator of RH for equisetum. The Δ^′17^O value of average phytolith is insensitive to RH. The simple linear relationship between RH and Δ^′17^O (2) is an oversimplification of a complex process of plant evaporation and changing conditions of growth. The relationship between RH and Δ^′17^O will vary for different plant types.
